# Supplementary material for: Phloroglucinol α-Pyrones from Helichrysum: A Review on Structural Diversity, Plant Distribution and Isolation
Source: Plants (Basel). 2025 Nov 12;14(22):3460. doi: 10.3390/plants14223460 (PMC12656563; doi:10.3390/plants14223460)
Supplement: Supplementary file 1 [file plants-14-03460-s001.zip › plants-3972141-supplementary.pdf]

# Supplementary material for review article “Phloroglucinol-α-Pyrones from *Helichrysum*: Structural Diversity, Plant Distribution and Isolation”

***Table S1.*** *Molecular characteristics of natural α-pyrones from Helichrysum.*

| Compound name | Molecular formula | Exact mass (Da) | Structure | SMILES | Subclass | Ref. |
|---|---|---|---|---|---|---|
| **3,5-dimethyl-4-hydroxy-6-isopropyl alphapyrone (1)** | $C_{10}H_{14}O_3$ | 182.0943 | 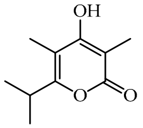 | O=C1C(C)=C(O)C(C)=C(C(C)C)O1 | Monopyrones | [16] |
| **3,5-dimethyl-4-(methoxy)-6-isopropyl alphapyrone (2)** | $C_{11}H_{16}O_3$ | 196.1099 | 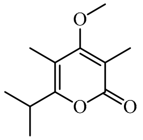 | O=C1C(C)=C(OC)C(C)=C(C(C)C)O1 | Monopyrones | [16] |
| **Micropyrone (3)** | $C_{14}H_{20}O_4$ | 252.1362 | 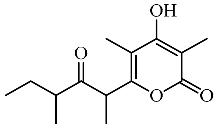 | CCC(C)C(=O)C(C)C1=C(C(=C(C(=O)O1)C)O)C | Monopyrones | [8,9,14,17] |
| **Helipyrone C (Bisnorhelipyrone) (4)** | $C_{15}H_{16}O_6$ | 292.0947 | 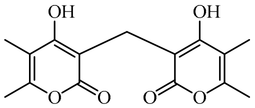 | CC1=C(C)C(O)=C(CC2=C(O)C(C)=C(C)OC2=O)C(O1)=O | Dipyrones | [5,11,19,20] |
| **Helipyrone B (Norhelipyrone) (5)** | $C_{16}H_{18}O_6$ | 306.1103 | 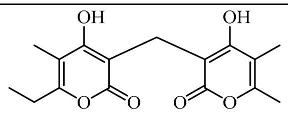 | CC1=C(C)C(O)=C(CC2=C(O)C(C)=C(CC)OC2=O)C(O1)=O | Dipyrones | [5,11,19,20] |
| **Helipyrone A (6)** | $C_{17}H_{20}O_6$ | 320.126 | 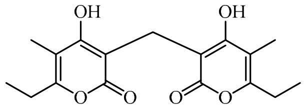 | CCC1=C(C(=C(C(=O)O1)CC2=C(C(=C(OC2=O)CC)C)O)O)C | Dipyrones | [5,7-9,11-13,17-20] |
| **Helitalone A (7)** | $C_{22}H_{30}O_5$ | 374.2093 | 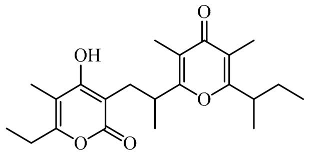 | O=C1C(CC(C2=C(C(C(C)=C(O2)C(CC)C)=O)C)C)=C(C(C)=C(O1)CC)O | Dipyrones | [14] |

| | | | | | | |
|---|---|---|---|---|---|---|
| **Norauricepyrone (8)** | $C_{20}H_{24}O_7$ | 376.1522 | 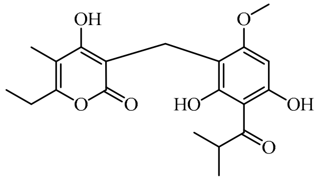 | OC1=C(CC(C2=O)=C(C(C)=C(O2)CC)O)C(OC)=CC(O)=C1C(C(C)C)=O | 3-NA PGs | [16] |
| **Methyl-norauricepyrone (9)** | $C_{21}H_{26}O_7$ | 390.1673 | 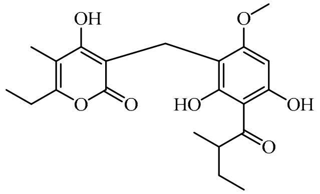 | OC1=C(CC(C2=O)=C(C(C)=C(O2)CC)O)C(OC)=CC(O)=C1C(C(CC)C)=O | 3-NA PGs | [16] |
| **Arzanol (10)** | $C_{22}H_{26}O_7$ | 402.1678 | 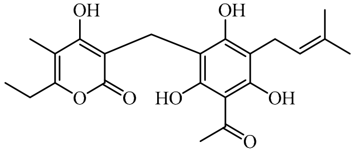 | CCC1=C(C)C(O)=C(CC2=C(O)C(C(C)=O)=C(O)C(C/C=C(C)\C)=C2O)C(O1)=O | 3-prenyl PGs | [8,9,13,14,17] |
| **Arenol B (11)** | $C_{23}H_{28}O_7$ | 416.1835 | 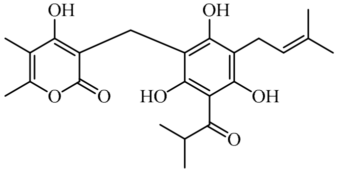 | O=C1C(CC2=C(C(C/C=C(C)\C)=C(C(C(C(C)C)=O)=C2O)O)O)=C(C(C)=C(O1)C)O | 3-prenyl PGs | [11] |
| **Helitalone B (12)** | $C_{23}H_{28}O_7$ | 416.1835 | 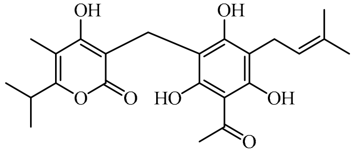 | O=C1C(CC2=C(C(C/C=C(C)\C)=C(C(C(C)=O)=C2O)O)O)=C(C(C)=C(O1)C(C)C)O | 3-prenyl PGs | [14] |
| **6-O-Desmethylauricepyrone (13)** | $C_{24}H_{30}O_7$ | 430.1991 | 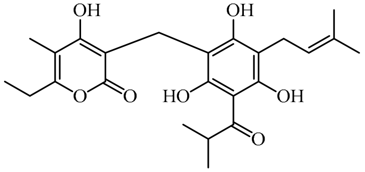 | O=C1C(CC2=C(C(C/C=C(C)\C)=C(C(C(C(C)C)=O)=C2O)O)O)=C(C(C)=C(O1)CC)O | 3-prenyl PGs | [7] |
| **Arenol C (14)** | $C_{24}H_{30}O_7$ | 430.1991 | 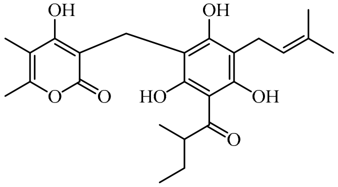 | O=C1C(CC2=C(C(C/C=C(C)\C)=C(C(C(C(C)CC)=O)=C2O)O)O)=C(C(C)=C(O1)C)O | 3-prenyl PGs | [11] |

| | | | | | | |
|---|---|---|---|---|---|---|
| **3-prenyl noraauricepyrone (15)** | $C_{24}H_{30}O_7$ | 430.1992 | 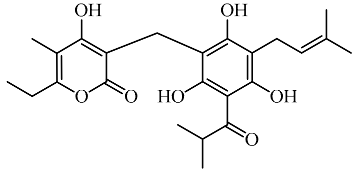 | O=C1C(CC2=C(C(C/C=C(C)\C)=C(C(C(C(C)C)=O)=C2O)O)O)=C(C(C)=C(O1)CC)O | 3-prenyl PGs | [11] |
| **23-Methyl-6-O-desmethylauricepyrone (16)** | $C_{25}H_{32}O_7$ | 444.2148 | 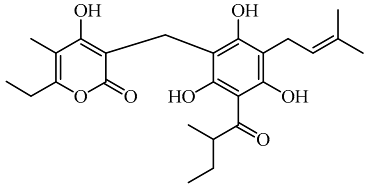 | O=C1C(CC2=C(C(C/C=C(C)\C)=C(C(C(C(C)CC)=O)=C2O)O)O)=C(C(C)=C(O1)CC)O | 3-prenyl PGs | [7,11,16] |
| **Heliarzanol (17)** | $C_{24}H_{30}O_8$ | 446.1941 | 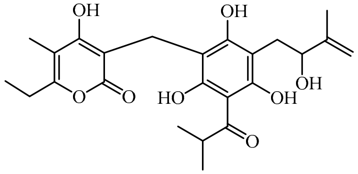 | O=C1C(CC2=C(O)C(CC(O)C(C)=C)=C(O)C(C(C(C)C)=O)=C2O)=C(O)C(C)=C(CC)O1 | 3-prenyl PGs | [9] |
| **23-ethyl-6-O-desmethyl-auricepyrone (18)** | $C_{26}H_{34}O_7$ | 458.2304 | 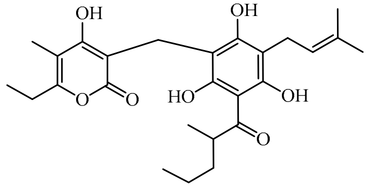 | OC1=C(CC2=C(O)C(C)=C(CC)OC2=O)C(O)=C(C(C(C)CCC)=O)C(O)=C1C/C=C(C)/C | 3-prenyl PGs | [16] |
| **Auricepyrone (19)** | $C_{25}H_{32}O_7$ | 444.2148 | 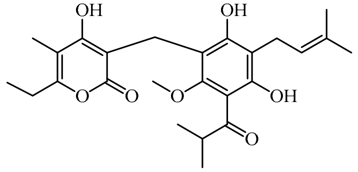 | O=C1C(CC2=C(C(C/C=C(C)\C)=C(C(C(C(C)C)=O)=C2OC)O)O)=C(C(C)=C(O1)CC)O | 3-prenyl methoxy PGs | [6,7,16] |
| **23-Methylauricepyrone (20)** | $C_{26}H_{34}O_7$ | 458.2304 | 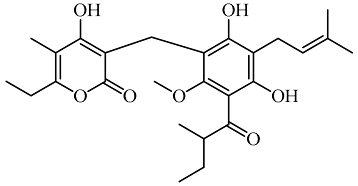 | O=C1C(CC2=C(O)C(C/C=C(C)\C)=C(O)C(C(C(CC)C)=O)=C2OC)=C(O)C(C)=C(CC)O1 | 3-prenyl methoxy PGs | [6,7] |

| | | | | | | |
|---|---|---|---|---|---|---|
| **23-ethyl-6-O-desmethyl-4-O-methylauricepyrone (21)** | $C_{27}H_{36}O_7$ | 472.2461 | 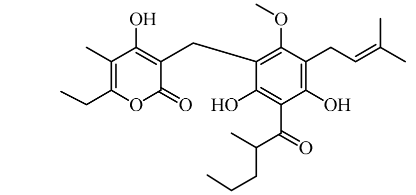 | OC1=C(C/C=C(C)/C)C(OC)=C(CC2=C(O)C(C)=C(CC)OC2=O)C(O)=C1C(C(C)CCC)=O | 3-prenyl methoxy PGs | [16] |
| **18,18-bis-desmethyl Achyroclinopyrone C (22)** | $C_{26}H_{32}O_7$ | 456.2148 | 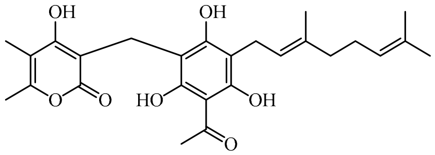 | CC(C1=C(O)C(CC2=C(O)C(C)=C(C)OC2=O)=C(O)C(C/C=C(CC/C=C(C)\C)\C)=C1O)=O | 3-geranyl PGs | [13,19,29] |
| **18,18-bis-desmethyl Achyroclinopyrone A (23)** | $C_{27}H_{34}O_7$ | 470.2304 | 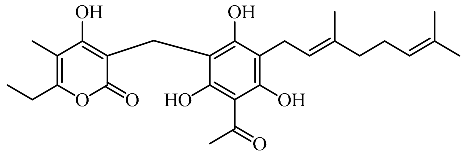 | O=C1C(CC2=C(O)C(C/C=C(CC/C=C(C)/C)\C)=C(O)C(C(C)=O)=C2O)=C(O)C(C)=C(CC)O1 | 3-geranyl PGs | [19,29] |
| **8'-methyl-18,18-bis-desmethyl Achyroclinopyrone A (24)** | $C_{28}H_{36}O_7$ | 484.2461 | 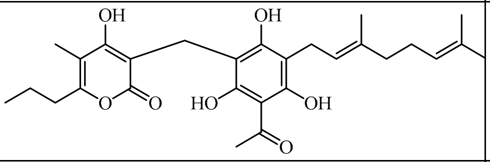 | CC(C1=C(O)C(CC2=C(O)C(C)=C(CCC)OC2=O)=C(O)C(C/C=C(CC/C=C(C)\C)\C)=C1O)=O | 3-geranyl PGs | [29] |
| **Achyroclinopyrone C (25)** | $C_{28}H_{36}O_7$ | 484.2461 | 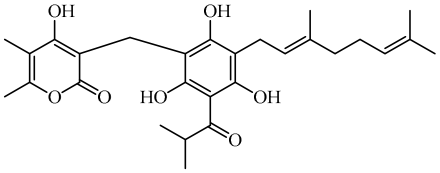 | CC(C)C(C1=C(O)C(CC2=C(O)C(C)=C(C)OC2=O)=C(O)C(C/C=C(CC/C=C(C)\C)\C)=C1O)=O | 3-geranyl PGs | [11] |
| **Achyroclinopyrone A (26)** | $C_{29}H_{38}O_7$ | 498.2617 | 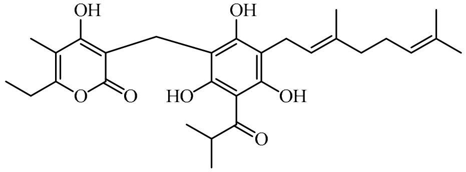 | CC(C)C(C1=C(O)C(CC2=C(O)C(C)=C(CC)OC2=O)=C(O)C(C/C=C(CC/C=C(C)\C)\C)=C1O)=O | 3-geranyl PGs | [11] |
| **Achyroclinopyrone D (27)** | $C_{29}H_{38}O_7$ | 498.2617 | 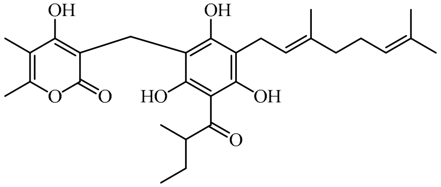 | O=C1C(CC2=C(O)C(C/C=C(CC/C=C(C)/C)\C)=C(O)C(C(C(CC)C)=O)=C2O)=C(O)C(C)=C(C)O1 | 3-geranyl PGs | [11] |

| | | | | | | |
|---|---|---|---|---|---|---|
| **Achyroclinopyrone B (28)** | $C_{30}H_{40}O_7$ | 512.2774 | 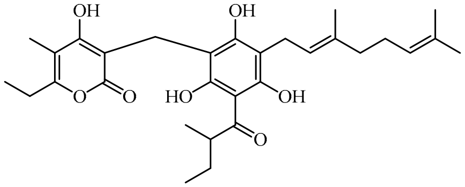 | O=C1C(CC2=C(O)C(C/C=C(CC/C=C(C)/C)\C)=C(O)C(C(C(CC)C)=O)=C2O)=C(O)C(C)=C(CC)O1 | 3-geranyl PGs | [11] |
| **Arenol A (29)** | $C_{21}H_{24}O_7$ | 388.1522 | 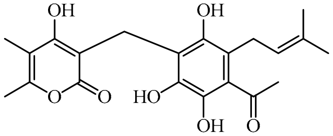 | O=C1C(CC2=C(O)C(C/C=C(C)\C)=C(C(C)=O)C(O)=C2O)=C(O)C(C)=C(C)O1 | 2-prenyl PGs | [15,20] |
| **Homoarenol (30)** | $C_{22}H_{26}O_7$ | 402.1678 | 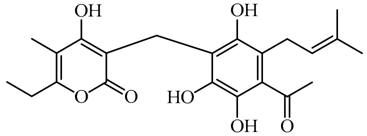 | O=C1C(CC2=C(C(C/C=C(C)\C)=C(C(O)=C2O)C(C)=O)O)=C(C(C)=C(O1)CC)O | 2-prenyl PGs | [15,20] |
| **Helichrytalicine B (31)** | $C_{19}H_{24}N_2O_6$ | 376.1634 | 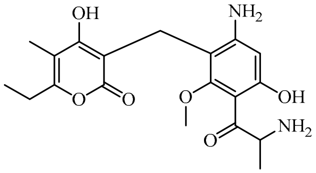 | O=C1C(CC2=C(N)C=C(O)C(C(C(N)C)=O)=C2OC)=C(O)C(C)=C(CC)O1 | Amino PGs | [12] |
| **Helichrytalicine A (32)** | $C_{20}H_{26}N_2O_6$ | 390.1791 | 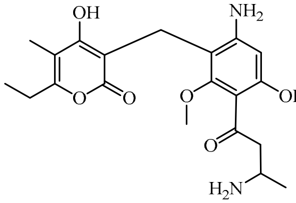 | O=C1C(CC2=C(N)C=C(O)C(C(CC(N)C)=O)=C2OC)=C(O)C(C)=C(CC)O1 | Amino PGs | [12] |
| **Plicatipyrone (33)** | $C_{22}H_{26}O_8$ | 418.1628 | 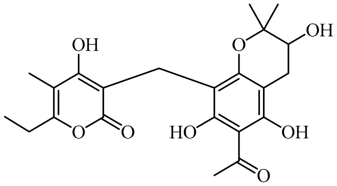 | CC(C(O)=C(C(O1)=O)CC2=C3C(CC(C(C)(O3)C)O)=C(C(C(C)=O)=C2O)O)=C1CC | Benzofuranes | [7,20] |

| | | | | | | |
|---|---|---|---|---|---|---|
| **Isobutyryl-helichromenopyrone (34)** | $C_{24}H_{28}O_7$ | 428.1835 | 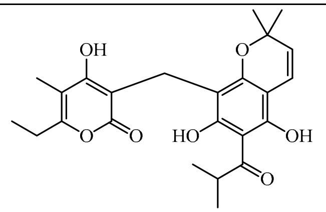 | CC(OC1=C2CC3=C(C(C)=C(OC3=O)CC)O)(C=CC1=C(O)C(C(C(C)C)=O)=C2O)C | Benzofuranes | [16] |
| **methylbutyryl-helichromenopyrone (35)** | $C_{25}H_{30}O_7$ | 442.1991 | 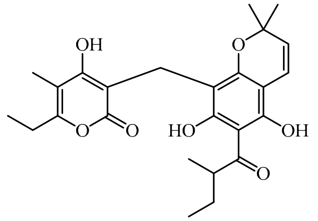 | CC(OC1=C2CC3=C(C(C)=C(OC3=O)CC)O)(C=CC1=C(O)C(C(C(C)CC)=O)=C2O)C | Benzofuranes | [16] |
| **Italipyrone (36)** | $C_{22}H_{24}O_7$ | 400.1522 | 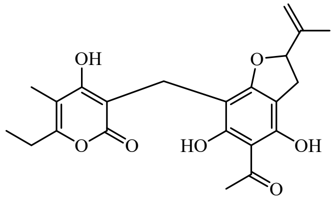 | OC1=C2C(OC(C(C)=C)C2)=C(CC3=C(O)C(C)=C(CC)OC3=O)C(O)=C1C(C)=O | Benzopyranes | [7,20] |
| **22-Methyl-22-ethyl-italipyrone (37)** | $C_{25}H_{30}O_7$ | 442.1991 | 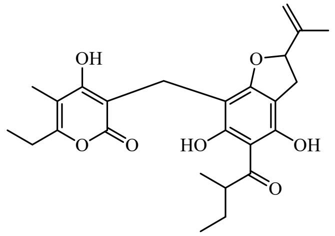 | OC1=C2C(OC(C(C)=C)C2)=C(CC3=C(O)C(C)=C(CC)OC3=O)C(O)=C1C(C(CC)C)=O | Benzopyranes | [7,16] |
| **22-Methyl-22-propyl-italipyrone (38)** | $C_{26}H_{32}O_7$ | 456.2148 | 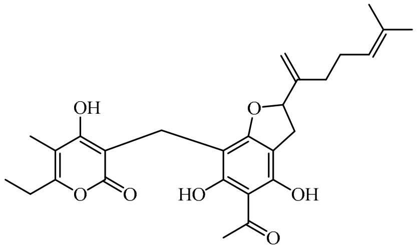 | OC1=C2C(OC(C(C)=C)C2)=C(CC3=C(O)C(C)=C(CC)OC3=O)C(O)=C1C(C(CCC)C)=O | Benzopyranes | [7,16] |

| | | | | | | |
|---|---|---|---|---|---|---|
| **20-(3,3′-Dimethylallyl)-italipyrone (39)** | $C_{27}H_{32}O_7$ | 468.2148 | 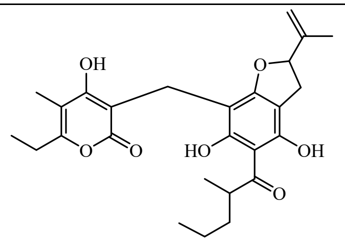 | OC1=C2C(OC(C2)C(CC/C=C(C)\C)=C)=C(C(O)=C1C(C)=O)CC3=C(C(C)=C(OC3=O)CC)O | Benzopyranes | [7] |
| **Helicyclol (40)** | $C_{28}H_{34}O_7$ | 482.2304 | 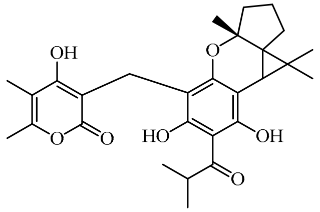 | CC1=C(C(O)=C(C(O1)=O)CC2=C3C(C(C4(C)C)C4(CCC5)[C@@]5(C)O3)=C(C(C(C(C)C)=O)=C2O)O)C | Benzopyranes | [11] |
| **Cycloarzanol C (41)** | $C_{21}H_{22}O_7$ | 386.1365 | 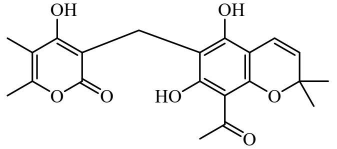 | CC1=C(C)C(O)=C(CC2=C(C3=C(OC(C)(C=C3)C)C(C(C)=O)=C2O)O)C(O1)=O | Chromane PGs | [11] |
| **Helicepyrone (42)** | $C_{29}H_{36}O_7$ | 496.2461 | 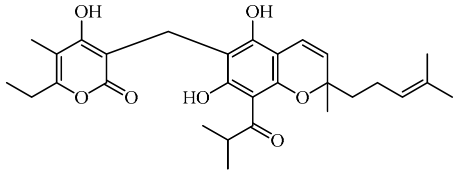 | CC(C(O)=C1CC2=C(C3=C(OC(C)(C=C3)CC/C=C(C)/C)C(C(C(C)C)=O)=C2O)O)=C(CC)OC1=O | Chromane PGs | [11] |
| **Italidipyrone (43)** | $C_{28}H_{32}O_{10}$ | 528.1995 | 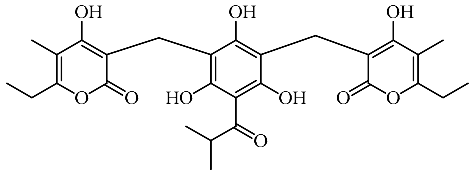 | O=C1C(CC2=C(O)C(CC3=C(O)C(C)=C(CC)OC3=O)=C(O)C(C(C(C)C)=O)=C2O)=C(O)C(C)=C(CC)O1 | Hetero-trimer PGs | [7,11] |
| **23-Methyl-italidipyrone (44)** | $C_{29}H_{34}O_{10}$ | 542.2152 | 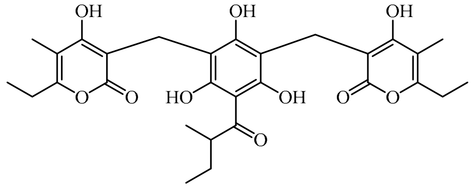 | O=C1C(CC2=C(O)C(CC3=C(O)C(C)=C(CC)OC3=O)=C(O)C(C(C(CC)C)=O)=C2O)=C(O)C(C)=C(CC)O1 | Hetero-trimer PGs | [7,11] |

| | | | | | | |
|---|---|---|---|---|---|---|
| **Helispiroketal A (45)** | $C_{20}H_{22}O_6$ | 358.1416 | 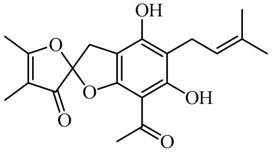 | OC1=C(C/C=C(C)/C)C(O)=C(C(C)=O)C2=C1CC3(C(C(C)=C(C)O3)=O)O2 | Spiroketals | [11] |
| **Helispiroketal B (46)** | $C_{21}H_{24}O_6$ | 372.1573 | 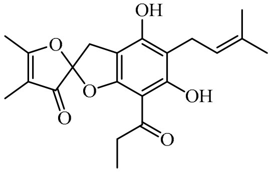 | OC1=C(C/C=C(C)/C)C(O)=C(C(CC)=O)C2=C1CC3(C(C(C)=C(C)O3)=O)O2 | Spiroketals | [11] |
| **Helispiroketal F (47)** | $C_{21}H_{24}O_6$ | 372.1573 | 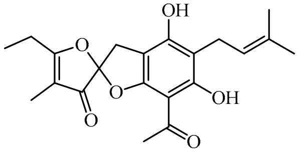 | OC1=C(C/C=C(C)/C)C(O)=C(C(C)=O)C2=C1CC3(C(C(C)=C(CC)O3)=O)O2 | Spiroketals | [11] |
| **Helispiroketal E (48)** | $C_{22}H_{26}O_6$ | 386.1729 | 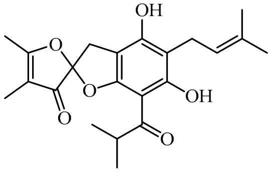 | OC1=C(C/C=C(C)/C)C(O)=C(C(C(C)C)=O)C2=C1CC3(C(C(C)=C(C)O3)=O)O2 | Spiroketals | [11] |
| **Helispiroketal C (49)** | $C_{23}H_{28}O_6$ | 400.1886 | 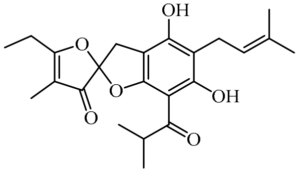 | OC1=C(C/C=C(C)/C)C(O)=C(C(C(C)C)=O)C2=C1CC3(C(C(C)=C(CC)O3)=O)O2 | Spiroketals | [11] |
| **Helispiroketal D (50)** | $C_{23}H_{28}O_6$ | 400.1886 | 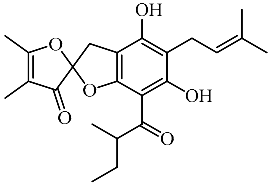 | OC1=C(C/C=C(C)/C)C(O)=C(C(C(CC)C)=O)C2=C1CC3(C(C(C)=C(C)O3)=O)O2 | Spiroketals | [11] |
| **Helispiroketal H (51)** | $C_{25}H_{30}O_6$ | 426.2042 | 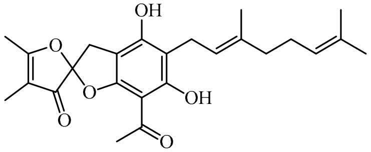 | OC1=C(C/C=C(C)/CC/C=C(C)/C)C(O)=C(C(C)=O)C2=C1CC3(C(C(C)=C(C)O3)=O)O2 | Spiroketals | [11] |

| | | | | | | |
|---|---|---|---|---|---|---|
| **Helispiroketal G (52)** | $C_{27}H_{34}O_6$ | 454.2355 | 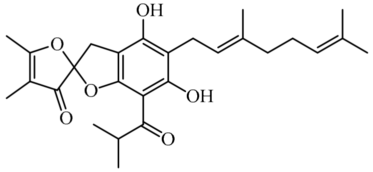 | OC1=C(C/C=C(C)/CC/C=C(C)/C)C(O)=C(C(C(C)C)=O)C2=C1CC3(C(C(C)=C(C)O3)=O)O2 | Spiroketals | [11] |

# References


5. Vrkcoč, J.; Dolejš, L.; Buděšínský, M. Methylene-bis-2H-pyran-2-ones and phenolic constituents from the root of *Helichrysum arenarium*. *Phytochemistry* **1975**, *14*, 1383-1384.
6. Bohlmann, F.; Zdero, C. Neue phloroglucin-derivate aus *Helichrysum*-arten. *Phytochemistry* **1980**, *19*, 153-155.
7. Hänsel, R.; Cybulski, E.-M.; Çubukçu, B.; Meriçli, A.H.; Bohlmann, F.; Zdero, C. Neue pyron-derivate aus *Helichrysum*-arten. *Phytochemistry* **1980**, *19*, 639-644.
8. Appendino, G.; Ottino, M.; Marquez, N.; Bianchi, F.; Giana, A.; Ballero, M.; Sterner, O.; Fiebich, B.L.; Munoz, E. Arzanol, an anti-inflammatory and anti-HIV-1 phloroglucinol α-pyrone from *Helichrysum italicum* ssp. *microphyllum*. *Journal of natural products* **2007**, *70*, 608-612.
9. Taglialatela-Scafati, O.; Pollastro, F.; Chianese, G.; Minassi, A.; Gibbons, S.; Arunotayanun, W.; Mabebie, B.; Ballero, M.; Appendino, G. Antimicrobial phenolics and unusual glycerides from *Helichrysum italicum* subsp. *microphyllum*. *Journal of natural products* **2013**, *76*, 346-353.
11. Akaberi, M.; Danton, O.; Tayarani-Najaran, Z.; Asili, J.; Iranshahi, M.; Emami, S.A.; Hamburger, M. HPLC-Based Activity Profiling for Antiprotozoal Compounds in the Endemic Iranian Medicinal Plant *Helichrysum oocephalum*. *Journal of Natural Products* **2019**, *82*, 958-969, doi:10.1021/acs.jnatprod.8b01031.
12. D'Abrosca, B.; Buommino, E.; Caputo, P.; Scognamiglio, M.; Chambery, A.; Donnarumma, G.; Fiorentino, A. Phytochemical study of *Helichrysum italicum* (Roth) G. Don: Spectroscopic elucidation of unusual amino-phlorogucinols and antimicrobial assessment of secondary metabolites from medium-polar extract. *Phytochemistry* **2016**, *132*, 86-94, doi:https://doi.org/10.1016/j.phytochem.2016.09.012.
13. Les, F.; Venditti, A.; Cásedas, G.; Frezza, C.; Guiso, M.; Sciubba, F.; Serafini, M.; Bianco, A.; Valero, M.S.; López, V. Everlasting flower (*Helichrysum stoechas M*oench) as a potential source of bioactive molecules with antiproliferative, antioxidant, antidiabetic and neuroprotective properties. *Industrial crops and products* **2017**, *108*, 295-302.
14. Werner, J.; Ebrahim, W.; Özkaya, F.C.; Mándi, A.; Kurtán, T.; El-Neketi, M.; Liu, Z.; Proksch, P. Pyrone derivatives from *Helichrysum italicum*. *Fitoterapia* **2019**, *133*, 80-84.
15. Vrkoč, J.; Dolejš, L.; Sedmera, P.; Vašíčková, S.; Šorm, F. The structure of arenol and homoarenol, α-pyrone derivatives from *Helichrysum arenarium* (L.) moench. *Tetrahedron Letters* **1971**, *12*, 247-250, doi:https://doi.org/10.1016/S0040-4039(01)96410-X.
16. Jakupovic, J.; Kuhnke, J.; Schuster, A.; Metwally, M.; Bohlmann, F. Phloroglucinol derivatives and other constituents from South African *Helichrysum* species. *Phytochemistry* **1986**, *25*, 1133-1142.

17. Rosa, A.; Deiana, M.; Atzeri, A.; Corona, G.; Incani, A.; Melis, M.P.; Appendino, G.; Dessì, M.A. Evaluation of the antioxidant and cytotoxic activity of arzanol, a prenylated α-pyrone–phloroglucinol etherodimer from *Helichrysum italicum* subsp. *microphyllum*. *Chemico-Biological Interactions* **2007**, *165*, 117-126.

18. Opitz, L.; Hänsel, R. Helipyron, ein methylen-bis-triacetsäurelacton aus Helichrysum italicum. *Tetrahedron Letters* **1970**, *11*, 3369-3370, doi:https://doi.org/10.1016/S0040-4039(01)98478-3.
19. Lavault, M.; Richomme, P. Constituents of *Helichrysum stoechas* variety olonnense. *Chemistry of natural compounds* **2004**, *40*.
20. Rios, J.; Recio, M.; Villar, A. Isolation and identification of the antibacterial compounds from *Helichrysum stoechas*. *Journal of Ethnopharmacology* **1991**, *33*, 51-55.
29. Tomás-Lorente, F.; Iniesta-Sanmartín, E.; Tomás-Barberán, F.A.; Trowitzsch-Kienast, W.; Wray, V. Antifungal phloroglucinol derivatives and lipophilic flavonoids from *Helichrysum decumbens*. *Phytochemistry* **1989**, *28*, 1613-1615.
